# Beneficial Effects of Spirulina Aqueous Extract on Vasodilator Function of Arteries from Hypertensive Rats

**DOI:** 10.1155/2020/6657077

**Published:** 2020-12-08

**Authors:** Diva M. Villalpando, Carlos M. Verdasco-Martín, Ignacio Plaza, Juan Gómez-Rivas, Fermín R de Bethencourt, Morris Villarroel, José L. García, Cristina Otero, Mercedes Ferrer

**Affiliations:** ^1^Departamento de Fisiología, Facultad de Medicina, Universidad Autónoma de Madrid, Madrid, Spain; ^2^Departamento de Biocatálisis, Instituto de Catálisis y Petroleoquímica, Consejo Superior de Investigaciones Científicas, Madrid, Spain; ^3^Departamento de Producción Agraria, ETSIAAB, Universidad Politécnica de Madrid, Madrid, Spain; ^4^Servicio de Urología, Hospital Universitario La Paz, Madrid, Spain; ^5^Instituto de Investigación Hospital Universitario La Paz (IdiPAZ), Madrid, Spain; ^6^Centro de Investigaciones Biológicas Margarita Salas, Biotecnología Medioambiental, Consejo Superior de Investigaciones Científicas, Madrid, Spain

## Abstract

Hypertension is a multifactorial disorder considered one of the major causes of premature death worldwide. This pathology is associated with vascular functional/structural alterations in which nitric oxide (NO) and oxygen reactive species participate. On the other hand, the use of microalgae extracts in the treatment of cardiovascular diseases is increasing. Based on the antioxidant and antihypertensive properties of Spirulina, this study aims to investigate the effect of an aqueous extract of Spirulina on the vasodilator function of the aorta from spontaneously hypertensive rats (SHR), analyzing the functional role of NO. For this, aortic segments from male SHR were divided into two groups, one control and the other exposed to an Spirulina aqueous extract (0.1% w/v, for 3 hours), to analyze (i) the production of NO, superoxide anion, and hydrogen peroxide; (ii) the vasodilator response induced by acetylcholine (ACh), by the NO donor and sodium nitroprusside (SNP), and by the K_ATP_ channel opener and pinacidil; and (iii) the expression of the p-Akt, p-eNOS, and HO-1 proteins. The results showed that the aqueous Spirulina extract (i) increased the production of NO, did not significantly modify that of superoxide, while decreased that of hydrogen peroxide; (ii) increased the vasodilatory responses induced by ACh, NPS, and pinacidil; and (iii) increased the expression of p-Akt and HO-1. These results suggest that incubation with the aqueous Spirulina extract improves the vascular function of arteries from SHR by increasing the release/bioavailability/function of NO. Increased K_ATP_ channel activation and expression of pAkt and HO-1 appear to be participating in these actions.

## 1. Introduction

Cardiovascular diseases represent the most common cause of death worldwide, and hypertension is one of the main risk factors for its development. This pathology is associated with vascular remodeling [1] that may be caused by an imbalance in the production and function of endothelial factors [2] which leads to increased vascular resistance. Among endothelial factors, nitric oxide (NO) plays a crucial role in vascular tone regulation through its vasodilator, antiproliferative, and antiaggregant actions [3]. It is well known that NO-induced vasodilation is mediated by increasing cyclic guanosine monophosphate (cGMP) levels and cGMP-dependent protein kinase (PKG) activation in vascular smooth muscles [4]. Likewise, NO, cGMP, and PKG are able to activate potassium channels and hyperpolarize the cell membrane [5, 6]. Thus, potassium channels are widely expressed in vascular tissue, and their role in vessel tone regulation has been well established [7]. The participation of hyperpolarizing mechanisms has gained relevance in physiopathological conditions matching with decreased NO bioavailability through the activation of calcium- and ATP-dependent potassium (K_Ca_ or K_ATP_, respectively) channels [8, 9]. In this regard, decreased NO bioavailability usually links to the increased production of reactive oxygen species (ROS), the latter being another hallmark of cardiovascular diseases including hypertension [10]. However, under pathophysiological conditions with increased oxidative stress, different homeostatic mechanisms can be activated. Thus, nuclear factor erythroid 2-related factor 2 (Nrf2) mediates the transcription of phase II antioxidant proteins responsible for the elimination of ROS [11]. Among the different target proteins of Nrf2, it should be noted that hemeoxygenase-1 (HO-1) has been recognized as one of the most important factors protecting vascular tissue from a prooxidant environment [12, 13].

In addition to the antihypertensive classical pharmacological therapy [14, 15], the consumption of natural compounds is increasing nowadays [16–19]. Recently, increasing attention has been paid to the use of microalgae since they contain various valuable natural compounds that have uses in the pharmaceutical, cosmetic, and nutraceutical industries [20, 21]. Among the microalgae, the filamentous cyanobacterium of the genus *Arthrospira* (e.g., *A. platensis*, *A. maxima*), commercially named as Spirulina, is referred to as a “superfood” with antioxidant-antihypertensive activities, cholesterol-controlling, and insulin resistance effects [22, 23]. Based on this information, the objective of this study was to analyze the effect of aqueous Spirulina extract on the vasodilator function of the aorta from spontaneously hypertensive rats (SHR) investigating the contribution of NO. Cell mediators (superoxide anion and hydrogen peroxide) and phosphorylation and/or expression of signalling proteins (eNOS, Akt, and HO-1) related to the production and/or bioavailability of NO were also studied.

## 2. Materials and Methods

### 2.1. Animals and Vascular Tissue Preparation

Male SHR rats, 5 months old, were selected by crossing Wistar-Kyoto rats with constitutive high blood pressure in the Animal Facility of the Universidad Autónoma de Madrid (UAM) (registration number EX-021U). All animal protocols were approved by the Research Ethics Committee of UAM according to directives 609/86 CEE and R.D. 233/88 of the Ministerio de Agricultura, Pesca y Alimentación of Spain (PROEX 202/16). The experiments were conducted in accordance with the published Guiding Principles in the Care and Use of Animals approved by the European Union directives 63/2010 UE and Spanish regulation RD53/2013. Systolic blood pressure was indirectly measured in awake animals by the tail-cuff method (Letica, Digital Pressure Meter, LE5000, Barcelona, Spain) before sacrifice. Rats were sacrificed by CO_2_ inhalation and subsequent decapitation; the aorta was carefully dissected out and placed in Krebs-Henseleit solution (KHS) at 4°C containing 115 mM NaCl, 2.5 mM CaCl_2_, 4.6 mM KCl, 1.2 mM KH_2_PO_4_, 1.2 mM MgSO_4_, 25 mM NaHCO_3_, and 11.1 mM glucose. The aorta, from SHR, was cleaned of adhering adipose and connective tissues, cut into rings of 4 mm in length, and divided into two groups according to the presence or absence of Spirulina: arteries exposed for 3 h to the aqueous Spirulina extract (0.1% w/v) and arteries incubated in KHS medium in which Spirulina was dissolved (control group) except for the experiments of NO release where the Spirulina extract was dissolved in 4-(2-hydroxyethyl)-1-piperazineethanesulfonic acid (HEPES) buffer containing 119 mM NaCl, 20 mM HEPES, 1.2 mM CaCl_2_, 4.6 mM KCl, 0.4 mM KH_2_PO_4_, 1 mM MgSO_4_, 5 mM NaHCO_3_, 5.5 mM glucose, and 0.15 mM Na_2_H_2_PO_4_.

### 2.2. Aqueous Extract of Spirulina

To obtain the *Spirulina* biomass, the *Arthrospira platensis* BIO1 strain, kindly supplied by Biodesma S.L., was used. *A. platensis* was grown in plastic bioreactors in a greenhouse at the Universidad Politécnica de Madrid (40.446353 N-3.738341 E), as previously described [24, 25]. Briefly, *A. platensis* was harvested when the culture reached approximately 1 g/L of dry weight. The harvest was performed from 9:00 to 10:30 hours when the protein content was higher [26]. Then, the cells were dried on a horizontal sheet at 50°C for approximately 4-6 h and kept in an opaque container at 4°C to prevent oxidation.

A polar Spirulina extract was prepared by solvent extraction according to the Bligh and Dyer method [27] after a prior step of Spirulina biomass sonication. Briefly, 50 mg of Spirulina biomass suspended in 1 mL of chlorophorm/methanol 1 : 2 (v/v) was submitted to 40 kHz sonication for 15 min at 25°C. The liquid phase was filtrated, and the recovered biomass was extracted with 0.5 mL of chlorophorm/methanol 1 : 2 (v/v). Next, the polar Spirulina biocomponents were separated by adding twice 0.6 mL of water with 0.58% NaCl to the chlorophorm/methanol phase. After gravimetric separation of phases for 24 h at 4°C, the aqueous phase was dried in a Buchi B480 rotary evaporator, weighed, and frozen at -70°C until use.

### 2.3. Vascular Reactivity

The method used for isometric tension recording has been described in full elsewhere [28]. Briefly, aortic segments were suspended in an organ bath containing 5 mL of KHS at 37°C, continuously bubbled with 95% O_2_-5% CO_2_ mixtures (pH 7.4). Two parallel stainless steel pins were introduced through the lumen of the vascular segment: one fixed to the bath wall and the other connected to a force transducer (Grass FTO3C; Grass Instruments Co., Quincy, MA, USA); this in turn was connected to a model 7D Grass polygraph. The aortic segments were subjected to a tension of 1 g which was readjusted every 15 min during a 90 min equilibration period before drug administration. After this, the vessels were exposed to 75 mM KCl to check the functional integrity. After a washout period, the viability of vascular endothelium was tested by the ability of 10 *μ*M ACh to relax precontracted segments with 0.1 *μ*M NA. Vessels were then washed with KHS to recover the basal tension. To investigate the effect of the aqueous Spirulina extract on the vasodilator response, separate aortic segments from SHR were incubated with the Spirulina extract (0.1% w/v) for 3 h (with changes every hour) before performing cumulative concentration-response curves to 0.1 nM-10 *μ*M ACh, to the NO donor 0.1 nM-10 *μ*M sodium nitroprusside (SNP), and to the K_ATP_ channel opener 0.1 *μ*M-10 *μ*M pinacidil in 0.1 *μ*M NA precontracted rings.

### 2.4. Release of Nitric Oxide

The release of NO was measured by using the fluorescent probe 4,5-diaminofluorescein (DAF-2, at 0.5 *μ*M) as previously reported [29, 30]. The fluorescence of the medium was measured at room temperature using a luminescence spectrometer LS50 (Perkin-Elmer Instruments) with the excitation wavelength set at 495 nm and emission wavelength at 515 nm. Also, blank measures were collected in the same way from the medium without mesenteric segments to subtract background emission.

### 2.5. Detection of Superoxide Anion

Hydroethidine, an oxidative fluorescent probe, was used to evaluate superoxide anion levels *in situ*, as previously described [29, 31, 32]. The tissue was also stained with the nuclear dye 4′,6-diamidino-2-phenylindole (DAPI, 10 *μ*g/mL). Segments were mounted on glad slides and imaged on a confocal microscope. Images were obtained with a LEICA (TCS ST2 DM IRE2) laser scanning confocal microscope to detect nuclei (405 nm excitation and 410-475 nm emission, for the DAPI dye) and oxidized hydroethidine (excitation 488 nm, emission 610 nm). Laser and image settings were unchanged for the acquisition of images from the three groups of rats. The photomicrographs show the intensity and location of hydroethidine, which reflects superoxide production, so that a comparison of these groups could be made. To analyze fluorescence intensity, the ImageJ Analysis Software (National Institutes of Health) was used. The amount of superoxide formation was expressed as the ratio between the fluorescence emitted by hydroethidine and that emitted by DAPI.

### 2.6. Production of Hydrogen Peroxide

The formation of hydrogen peroxide in aortic rings was measured by using a fluorescence H_2_O_2_ assay kit (Cayman Chemical) as previously reported [32]. Some assays were performed in the presence of catalase, an H_2_O_2_ scavenger, to ensure the specificity of the method. The fluorescence at 530 and 590 nm excitation and emission wavelengths, respectively, was registered in a 96-microplate reader (Multiskan Ascent, Labsystems). The protein content in the aortic samples was quantified by the bicinchoninic acid assay using the BCA™ Protein Assay Kit (Pierce). Data were expressed as nanomole per microgram of protein.

### 2.7. Western Blot Analysis of p-Akt, p-eNOS, and HO-1 Expression

Arterial segments were homogenated and processed to quantify protein concentration at 4°C in RIPA buffer containing phosphatase inhibitors and a cocktail of protease inhibitors. Proteins (20 *μ*g) were separated by SDS-PAGE gels and transferred to polyvinylidene difluoride (PVDF) membranes (Bio-Rad Immun-Blot® overnight at 4°C, 230 mA, using a Bio-Rad Mini Protean III system (Bio-Rad Laboratories, Hercules, CA, USA). Membranes were blocked with 5% (w/v) fat-free powdered milk or 5% (w/v) bovine serum albumin following the instructions of the antibodies' manufacturers and incubated overnight with mouse monoclonal antibody for phospho-eNOS (1:250dilution), purchased from Transduction Laboratories (Lexington, UK), anti-phospho-Akt (S473), or with rabbit polyclonal antibody for HO-1 (1 : 2000 dilution), purchased from Stresggen Bioreagents (Victoria, Canada). After washing, the membrane was incubated with the corresponding anti-immunoglobulin G conjugated to horseradish peroxidase (Amersham International Plc). The membrane was thoroughly washed, and the immunocomplexes were detected using an enhanced horseradish peroxidase/luminol chemiluminescence system (ECL Plus, Amersham International Plc, Little Chalfont, UK) and subjected to autoradiography (Hyperfilm ECL, Amersham International Plc). Signals on the immunoblot were quantified using a computer program (NIH Image V1.56). The same membrane was used to determine GAPDH expression, and the content of the latter was used to correct p-AKt, p-eNOS, and HO-1 expression in each sample, by means of a monoclonal antibody anti-GAPDH (1 : 5000 dilution, Sigma).

### 2.8. Data Analysis

Results are given as mean ± SEM (Standard Error of the Mean). The relaxation induced by ACh, SNP, or pinacidil was expressed as a percentage of initial contraction elicited by NA. Statistical analysis was performed by comparing the curves obtained in aortae from SHR rats after Spirulina extract incubation with that obtained in the absence of the extract by means of two-way analysis of variance (ANOVA). For NO, superoxide anion and hydrogen peroxide production and proteins expression statistical analysis was done using Student's *t*-test for unpaired experiments. A *p* value of less than 0.05 was considered significant. The statistical analysis and the elaboration of the graphs were carried out using the statistical program GraphPad PRISM® (Version 6.01).

### 2.9. Drugs and Chemicals

The drugs used were as follows: DAF-2, HE, L-NA hydrochloride, ACh chloride, potassium chloride, SNP, and pinacidil (Sigma-Aldrich). The stock solutions (10 mM) of drugs were prepared in distilled water, except for NA which was dissolved in NaCl (0.9%)-ascorbic acid (0.01% w/v) solution. These solutions were kept at -20°C, and appropriate dilutions were made in KHS on the day of the experiment.

## 3. Results

### 3.1. Animal Weight and Systolic Blood Pressure

The body weight (315 ± 9.5 g) and systolic blood pressure of the SHR (162 ± 4.7 mmHg) were similar to those reported in previous studies [33–35].

### 3.2. Vascular Reactivity

**Figure 1:**
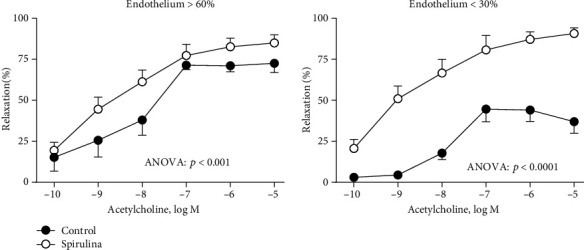
Effect of the aqueous Spirulina extract incubation on the concentration-response curve to acetylcholine in aortic segments, with different amounts of functional endothelium, from SHR. Results (means ± SEM) are represented as the percentage of inhibition of the contraction elicited by 0.1 *μ*M noradrenaline. Number of animals: 5-7. The statistical significance is indicated in the corresponding graphs.

In NA-precontracted arterial segments, the vasodilator response induced by ACh (0.1 nM-10 *μ*M) was increased after Spirulina extract incubation (Figure 1). A detailed analysis showed that the Spirulina-induced increase of the vasodilatory action of Ach was greater in vessels with less than 30% of endothelium than in those with above 60% endothelium (Figures 1(a), 1(b)).

**Figure 2:**
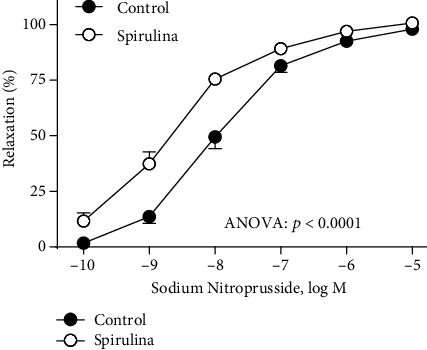
Effect of the aqueous Spirulina extract incubation on the concentration-response curve to the NO donor sodium nitroprusside in aortic segments from SHR. Results (means ± SEM) are presented as a percentage of inhibition of the contraction elicited by 0.1 *μ*M noradrenaline. Number of animals: 5-7. The statistical significance is indicated in the graph.

To analyze the possible action of Spirulina on the sensitivity of smooth muscle cells to NO, the vasodilator response induced by the NO donor, SNP, was studied. After incubation with Spirulina extract, the vasodilator response elicited by SNP (0.1 nM-10 *μ*M) was increased (Figure 2).

**Figure 3:**
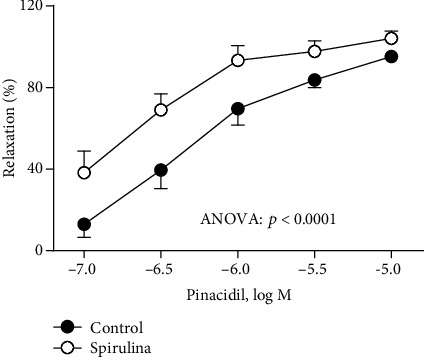
Effect of the aqueous Spirulina extract incubation on the concentration-response curve to the K_ATP_ channel opener pinacidil in aortic segments from SHR. Results (means ± SEM) are represented as the percentage of inhibition of the contraction elicited by 0.1 *μ*M noradrenaline. Number of animals: 5-7. The statistical significance is indicated in the graph.

Due to the involvement of K_ATP_ in the vasodilator responses, the effect of the Spirulina extract on the function of the K_ATP_ channels was analyzed. Concentration-response curves to the K_ATP_ channel opener pinacidil (0.1 *μ*M-10 *μ*M) were analyzed in 0.1 *μ*M NA precontracted aortic rings from SHR ± Spirulina extract. The results showed that the vasodilator response to pinacidil was enhanced after incubation with Spirulina extract (Figure 3).

### 3.3. NO Release

**Figure 4:**
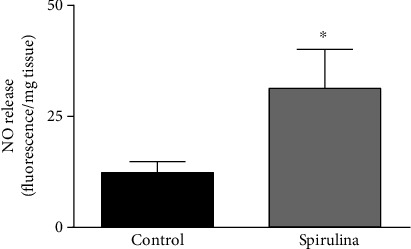
Effect of the aqueous Spirulina extract incubation on the basal release of NO in aortic segments from SHR. Results (means ± SEM) are expressed as arbitrary units of fluorescence (AU) per milligram of tissue. Number of animals: 5-7. ^∗^*p* < 0.05 compared with the control condition (in the absence of Spirulina extract).

After incubation with 4,5-diaminofluorescein, the fluorescence emitted was higher in arteries exposed to the aqueous extract of Spirulina with respect to the control condition (Figure 4).

### 3.4. Detection of Superoxide Anion

**Figure 5:**
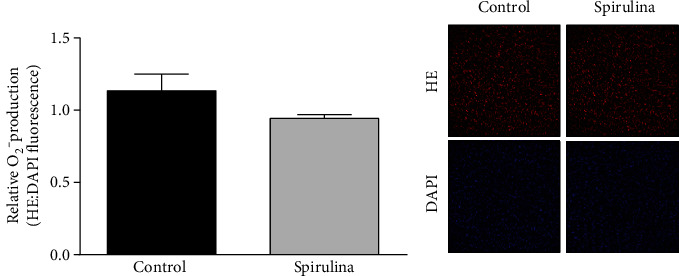
Effect of the aqueous Spirulina extract incubation on the production of superoxide anion in aortic segments from SHR. Representative confocal images showing in situ detection of superoxide anion in red and DAPI-nuclei staining in blue. Quantitative analysis of fluorescence is also shown. Results (means ± SEM) are expressed as the ratio between the fluorescence emitted by hydroethidine (HE) and that emitted by DAPI. Number of animals: 4.

In arteries from SHR, the incubation with Spirulina extract did not significantly modify the production of superoxide anion (Figure 5). The Spirulina extract did not modify the fluorescence emitted by DAPI.

### 3.5. Detection of Hydrogen Peroxide

**Figure 6:**
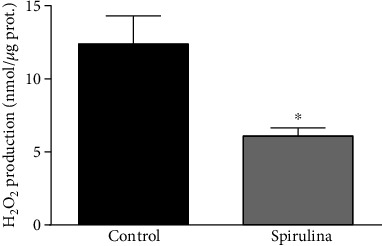
Effect of the aqueous Spirulina extract incubation on the production of hydrogen peroxide in aortic segments from SHR. Results (means ± SEM) are expressed as nanomoles of H_2_O_2_ per microgram of protein. Number of animals: 5. ^∗^*p* < 0.01 compared with the control condition (in absence of Spirulina extract).

In aortae from SHR, the incubation with the Spirulina extract reduced the levels of hydrogen peroxide (Figure 6).

### 3.6. Expression of p-eNOS, p-Akt, and HO-1

**Figure 7:**
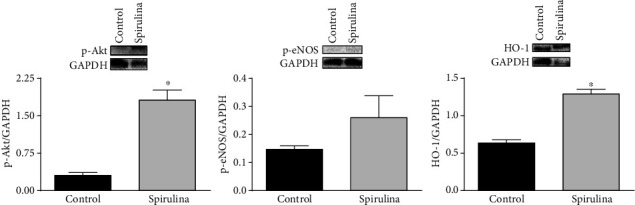
Representative western blot and densitometric analysis for the expression of p-Akt, p-eNOS, and HO-1 protein in aortic segments from SHR in the absence (control) or in the presence of the aqueous Spirulina extract. Results (means ± SEM) are expressed as the ratio between the signal for p-Akt, p-eNOS, or HO-1 protein and the signal for GAPDH. Number of animals: 3-5. ^∗^*p* < 0.01 compared with the control condition (in the absence of Spirulina extract).

The effect of Spirulina extract on the expression of p-Akt, p-eNOS, and HO-1 was analyzed in homogenates of aortae from SHR by using western blot analysis. The results show that the expression of p-Akt and HO-1 were increased, while that of p-eNOS was not statistically modified after Spirulina incubation (Figure 7).

## 4. Discussion

Overall, the present work shows that incubation with the aqueous extract of Spirulina improves the vasodilator response in arteries from hypertensive rats. Additionally, the increase in the NO release/bioavailability and in the activity of potassium channels seem to be mechanisms involved in the Spirulina-induced effect.

Hypertension has been described to compromise the vasodilator function by altering the release/function of vasodilator factors, among which NO and ROS are relevant players. Based on previous observations, the effect of the aqueous extract of Spirulina on the ACh-induced vasodilation was analyzed in arteries from SHR rats with different amounts of functional endothelium. The results show that in the aorta with functional endothelium above 60%, the Spirulina extract induced lesser increase in the ACh-induced response than in arteries with functional endothelium below 30%. These results indicate that aqueous Spirulina extract seems to act through endothelial-dependent and endothelial-independent mechanisms. Regarding endothelial-dependent mechanisms, one plausible mechanism would be increased NO release. Our results show that the release of NO was increased in arteries from SHR, as in previous studies [36, 37]. Additionally, since increased production of contractile prostanoids has been reported in hypertension [38], so the greater effect exerted by Spirulina could also be due to the decrease in the contractile factors as the endothelium is removed. However, the participation of mediators other than NO in the Spirulina-induced effect cannot be ruled out and will require further investigation.

Once the NO released was determined, the following step was to analyze the possible modification that Spirulina extract could exert on the sensitivity of the smooth muscle to the NO. The results show that the SNP-induced response was increased by the aqueous extract which appears to agree with studies describing an increased participation of NO in the responses induced by a different agonist after Spirulina treatment [36, 37]. Additionally, it is important to note that the greatest increment observed in the SNP-induced relaxation after Spirulina extract incubation was observed at low concentrations of SNP, which may be considered of physiological relevance. Although there is no specific information on the effect of Spirulina extract on the NO donor-induced vasodilation, the results described here indicate that Spirulina may also act through endothelium-independent mechanisms.

Taking into account that NO can hyperpolarize cell membranes by activating potassium channels and that changes in the activity of K_ATP_ channels have been implicated in the development of hypertension [39, 40], the vasodilator response to the K_ATP_ channel opener pinacidil was investigated. The results show that the pinacidil-induced relaxation was increased by the incubation with the aqueous Spirulina extract, which suggests that Spirulina extract could increase the activity of K_ATP_ channels. To date, the activation of potassium channels by Spirulina water-soluble components has not been reported, but it could be a plausible hypothesis as occurs with lipophilic components such as polyunsaturated fatty acids that are able to activate potassium channels [32, 41, 42]. The fact that the greatest effect was again observed at low concentrations of pinacidil points out the physiological relevance of Spirulina extract exposure, since the same profile in the effect of the aqueous extract on the three tested vasodilator responses was observed.

Interestingly, it has been reported that opening K_ATP_ channels increase the expression of p-Akt [43]. Since p-AKt is involved in the activation of eNOS, the expression of both proteins was also analyzed; the results showed that Spirulina extract increased the expression of p-Akt which can be responsible for the increased production of NO. However, Spirulina extract failed to significantly increase the expression of p-eNOS which suggests an increase of eNOS activity and/or a decrease of NO metabolism that would match with the increased NO release observed after incubating vessels with the Spirulina extract. Since the antioxidant property of Spirulina has been reported, an increase in the NO bioavailability cannot be ruled out. In this regard, it is important to note that in response to prooxidant environments, as occurs in hypertension, HO-1 is one of the earliest expressed proteins and its involvement in cardiovascular protection has been described [12, 13]. The fact that the Spirulina extract increases the expression of HO-1 would be in agreement with the antioxidant properties of the cyanobacteria, as has been described in glial cells [44]. Additionally, several investigations have reported that the increased expression of HO-1 induced by different natural products is mediated through p-AKt [45–48]. Therefore, the next step was to analyze the influence of the Spirulina extract on the formation of superoxide anion. The results, contrary to what was expected, show no statistical differences in the production of superoxide anion after incubation with the Spirulina extract. This result apparently disagrees with most of the studies describing an antioxidant effect of Spirulina [49–51]. One possible explanation is that higher concentration and/or incubation time of Spirulina was required to diminish the overproduction of superoxide, either by modulating NADPH oxidase and/or SOD activity. Interestingly, the choice of the cyanobacteria strain is also important, since differences in the synthesis of bioactive compounds, even in smaller quantities, may have an impact on health-promoting properties [52]. Another possibility could be that SOD was already activated as a compensatory mechanism in an attempt to eliminate the elevated superoxide production reported in different physiopathological conditions such as hypertension [53], atherosclerosis [54], and/or aging [55] and that the Spirulina extract could not induce further activation. Therefore, the content of hydrogen peroxide was analyzed, showing that the incubation with the Spirulina extract decreased the production of this particular ROS. This result agrees with most of the studies describing the general antioxidant properties of Spirulina [36, 37]. There are still remaining important questions about the differences between the properties of the isolated Spirulina extracts and the properties exerted on specific biological tissues, depending also on the particular pathophysiological condition of tissues. Overall, these considerations ensure future studies paying special attention to the Spirulina extract diet supplementation, since these experiments could report any potential improvement in different biochemical, cellular, and physiological parameters directly related to the maintenance of a healthy vascular function.

## 5. Conclusion

In summary, the current study describes some of the possible mechanisms involved in the effects induced by the Spirulina extract, paying special attention to the functional role of NO. Incubation with an aqueous Spirulina extract improved the vasodilator response of the aorta from hypertensive rats through the increased NO release/bioavailability and function, linked to the increased expression of p-Akt and HO-1. Also, the activation of K_ATP_ channels was involved in that effect.

## Data Availability

The data used to support the findings of this study are included within the article.
